# Risk factors of anastomotic leak in colorectal cancer: a multicentric study in a Latin American country

**DOI:** 10.3332/ecancer.2024.1696

**Published:** 2024-04-16

**Authors:** Sergio Luis Ramos Rodriguez, David Francisco Stein Montoro, Gabriel De la Cruz Ku, Consuelo del Rocio Luna Munoz, Cesar Ramon Razuri Bustamante

**Affiliations:** 1 Universidad Ricardo Palma, Lima 15033, Peru; 2 Universidad Cientifica del Sur, Lima 15067, Peru; 3 Hospital Nacional Edgardo Rebagliati Martins, Lima 15072, Peru; 4 Hospital Nacional Dos de Mayo, Lima 15003, Peru

**Keywords:** anastomotic leak, risk factors, colon cancer, colorectal surgery, colon resection (MESH)

## Abstract

**Introduction:**

The anastomotic leak (AL) is one of the most feared complications of colorectal surgery, since it is associated with a high rate of morbidity, mortality, length of hospital stay and cost of care. Our aim was to determine the risk factors associated with anastomosis leak in colorectal cancer patients who underwent surgical resection with anastomosis.

**Methods:**

A multicentre observational, analytical, retrospective and case-control study was carried out. For each case, two controls were included from three national hospitals from Lima, Peru during the period 2021–2022. To determine the degree of association, multivariate logistic regression model was carried out.

**Results:**

A total of 360 patients were included, 120 from each hospital. The mean age of the population was 68.03 ± 14.21 years old. The majority were 65 years old or older (66.1%), 52.8% were female, and 63.3% had clinical stage III. The 40% of the patients had albumin levels lower than 3.5 g/dL. Regarding the surgery, 96.4% were elective, 68.9% underwent open approach, and 80.8% had an operative time of more than 180 minutes. Most of them had right colon cancer (50.8%). In the multivariate analysis, a significant association was found with the age variable (OR = 2.48; 95%CI:1.24–4.97), clinical tumour level (OR = 2.71; 95%CI:1.34–5.48), American Society of Anesthesiologists (ASA) Score (OR = 3.23; 95%CI:1.10–9.50), preoperative serum albumin (OR = 22.2; 95%CI:11.5–42.9).

**Conclusion:**

The most important independent risk factors associated with AL among patients with colorectal cancer were pre-operative such as lower preoperative serum albumin levels, followed by a higher ASA Score, clinical-stage III-IV, and an age ≥65 years old.

## Introduction

Colorectal cancer is the third malignant neoplasm diagnosed worldwide with an incidence of approximately 1.93 million cases per year and 935, 173 deaths in 2022, being the second cause of mortality due to cancer, while in Peru, this is the third most diagnosed cancer [1]. One of the most life-threatening complications after undergoing oncologic resection is anastomotic leak (AL) the incidence of which varies between 1% and 19%, usually more frequent in the rectum [2, 3].

The AL is associated with higher morbidity, mortality, length of stay and health costs, with a high impact on the public health of the country due to the poor quality of life and assistance needs from the affected patients. Several studies have been performed to determine the risk factors of this complication to prevent or diagnose it in an early stage [4, 5]. Literature refers to some factors associated with AL such as male sex, age, tobacco, hypoalbuminemia, anaemia, previous abdominal surgeries, absence of ostomy to protect the anastomosis, operative time, type of anastomosis, radiotherapy, and tumour staging [6].

To date, there is scarce literature regarding this complication and its risk factors in a developing country and Latin population where a high percentage of the surgeries are open instead of laparoscopic, and most of the hospitals do not have enhanced recovery after surgery (ERAS) protocols. For instance, open surgery was the standard of care in the public health system at the beginning of the last decade; despite, there is trend to adopt the laparoscopic approach, there is still around 50%–80% of the cases open during the last 5 years [7–10]. Hence, our purpose is to identify the pre-, intra- and post-operative risk factors associated with AL from three national hospitals in the capital of Peru to allow the surgeons to make decisions on a daily basis and provide individualised surgical treatment to patients with colorectal cancer.

## Materials and methods

### Study design and population

We conducted a retrospective case control study of patients with pathology diagnosis of colorectal cancer who underwent surgery at three national hospitals from Lima, the capital of Peru: ‘Dos de Mayo’ National Hospital, ‘Edgardo Rebagliati Martins’ National Hospital, and ‘Alberto Sabogal Sologuren’ National Hospital between January 2021 and December 2022. The first hospital belongs to the public health insurance network of the Ministry of Health which is a national insurance offered to any Peruvian citizen and covers approximately 80% of the costs, while the second and third hospitals belong to the Ministry of Labour and Employment, which is an insurance only for employees from any national or independent job. Moreover, the leak rate per hospital was 6% for ‘Dos de Mayo’ National Hospital, 8% for ‘Alberto Sabogal Sologuren’ National Hospital, and 8% for ‘Alberto Sabogal Sologuren’ National Hospital. All of these hospitals are considered high volume of cases. Regarding the surgeons who performed the surgery, the three hospitals had one senior colorectal consultant, one junior colorectal surgeon, one junior general surgeon, but different number of residents; 10 residents at ‘Dos de Mayo’ National Hospital, 15 residents at ‘Alberto Sabogal Sologuren’ National Hospital, and 8 residents at ‘Alberto Sabogal Sologuren’ National Hospital; none of them had fellows in training.

The medical records were identified through the electronic health record system of the hospitals. Cases were defined as patients who were diagnosed with AL with imaging (computed tomography) reviewed by a licensed radiologist and colorectal surgeon until 30 days after surgery according to the consensus on the definition of colorectal anastomotic leakage: extravasation of endoluminal contrast, collection around the anastomosis, pre-sacral abscess adjacent to the anastomosis, air around the anastomosis, and pneumoperitoneum [11]. Controls were defined as patients without diagnosis of AL within 30 days after surgery, who were 18 years old or older, with pathological diagnosis of colorectal cancer, and who underwent either elective or emergency surgery. Exclusion criteria were as follows: patients who underwent neoadjuvant chemotherapy, incomplete medical records, patients who arrived at the emergency and were not admitted to the institution, presence of carcinomatosis, patients who underwent colostomy creation, palliative surgery, low anterior resection, or not creation of anastomosis, and those who died during the surgical intervention. The cases were selected according to the inclusion and exclusion criteria, to obtain an equal number of cases per hospital, if the number was higher than 40 patients, patient medical record numbers underwent simple random selection in Microsoft Excel. For each case, two controls were included. Controls were paired with cases according to the variables age and sex. Moreover, air leak test was performed in 55% of all the cases and controls, endoscopy or colonoscopy were not used, and all the patients who underwent leak test had a negative result intraoperatively.

### Variables

Our study included sociodemographic, pre-operative laboratory, and surgical characteristics. All the clinical data collection was obtained through a manual review of the medical records, these were reviewed by two of the authors (SLRR and DFSM). Variables included were age, sex, presence of comorbidities (hypertension, type 2 diabetes mellitus), tobacco use, alcohol intake, American Joint Committee on Cancer (AJCC) stage, American Society of Anesthesiologists (ASA) physical status classification system, pre-operative albumin, haemoglobin, creatinine, setting of the surgery (elective or emergency), surgical approach (laparoscopic or open), operative time, anastomosis technique (manual sewing or stapler), type of anastomosis (end to end, end to side, or side to side), intraoperative transfusion, tumour location (right, transverse, left colon, or rectum) and time to restart diet.

### Data analysis

Demographic, clinical, and surgical characteristics were reported with descriptive statistics. Quantitative variables were reported in mean and standard deviation, while quantitative variables that were dichotomised and qualitative variables were reported in frequencies and percentages. These characteristics were reported in general, according to the hospital that patients underwent surgery, and according to the presence of leak anastomotic. Chi-square test was used to assess the relation between characteristics and the presence of AL. Univariate and multivariate logistic regression analysis was performed to assess the factors associated to AL, this model was adjusted for age, sex, hypertension, type 2 diabetes mellitus, tobacco use, alcohol intake, AJCC stage, ASA score, pre-operative albumin, haemoglobin, creatinine, setting of the surgery, surgical approach, operative time, anastomosis technique, type of anastomosis, intraoperative transfusion, tumour location and time to restart diet. We report our outcomes adjusted odds ratios and 95% confidence intervals. A *p*-value lower than 0.05 was considered statistically significant. All analyses were performed in the software IBM SPSS Statistics version 29.0.

### Ethical statement

This study was approved by the Institutional review board of the ‘Universidad Ricardo Palma’ and all the hospitals ‘Dos de Mayo’ National Hospital, ‘Edgardo Rebagliati Martins’ National Hospital, and ‘Alberto Sabogal Sologuren’ National Hospital. Patients’ data were registered using codes and no personal information was abstracted to the dataset.

## Results

A total of 360 patients with colorectal cancer were included, 120 patients from each hospital; ‘Dos de Mayo’ National Hospital, ‘Edgardo Rebagliati Martins’ National Hospital, and ‘Alberto Sabogal Sologuren’ National Hospital. The mean age was 68.03 years (standard deviation: 14.21), 66.1% were 65 years old or older and 62.8% were females. Most of our population had higher AJCC stages III and IV (69.2%), while malnutrition based on albumin <3.5 g/dL and low haemoglobin <11 g/dL was found in 40% and 44.4% of the patients, respectively. The 91.9% had an ASA score of II. 96.4% of patients underwent elective surgery and 68.9% were open approach with hand sewing technique (61.1%) and side-to-side anastomosis (65.0%). Moreover, mostly of the cases lasted more than 180 minutes (80.8%). Intraoperative transfusion was given in 3.9% of patients. The most common location of the tumour was the right colon (50.8%), followed by the left colon (28.3%), rectum (13.9%) and transverse colon (6.9%). Furthermore, diet was started more than 3 days after surgery in 90.8% of patients Table 1.

When the three hospitals were compared, the hospital with national insurance had a higher population younger than 65 years old (*p* = 0.020), with a lower ASA score (*p* = 0.024) compared to the other two hospitals with employee insurance. Regarding the surgical characteristics, ‘Edgardo Rebagliati Martins’ National Hospital and ‘Alberto Sabogal Sologuren’ National Hospital performed more open procedures (*p* < 0.001) with lower operative time ≤180 minutes (*p* < 0.001) Table 2.

Patients who were 65 years or older (76.7% versus 60.8%, *p* = 0.003), a higher stage (82.5% versus 62.5%, *p* < 0.001), ASA score III versus II (13.% versus 5.4%, *p* = 0.013), preoperative albumin <3.5 g/dL (81.7% versus 19.2%, *p* < 0.001), and a tumour located in the rectum (24.2% versus 8.8%), were factors related to more frequency of AL Table 3.

In the univariate analysis, the factors associated with the presence of AL were age of 65 years old or older (OR = 2.11; 95%CI: 1.29–3.48; *p* = 0.003, AJCC stage III–IV versus. I–II (OR = 2.82; 95%CI: 1.65–4.85; *p* < 0.001), ASA score III versus II (OR = 2.68; 95%CI: 1.25–5.79; *p* < 0,05), prealbumin level lower than 3.5 g/dL (OR = 18.7; 95%CI: de 10.7–32.9; *p* < 0.001), and tumour location in the rectum versus right colon (OR = 3.57; 95%CI: 1.87–6.83; *p* < 0.001). While in the multivariate analysis, the independent factors associated to AL were age of 65 years old or older (OR = 2.48; 95%CI: de 1.24–4.97; *p* = 0.010), AJCC stage III–IV versus I–II (OR = 2.71; 95%CI: 1.34–5.48; *p* = 0.005), ASA score (OR = 3.23; 95%CI: 1.10–9.50; *p* = 0.032), and prealbumin level lower than 3.5 g/dL (OR = 22.2; 95%CI: 11.5–42.9; *p* < 0.001) Table 4.

## Discussion

AL after surgery for colorectal surgery has serious consequences for the quality of life of the patients affected and the health system [12–14]. Our multicentric study included three national hospitals from a developing Latin country with high health care needs and found that only preoperative characteristics of the patients were independent risk factors for AL such as pre-operative albumin level, followed by ASA score, higher tumour staging, and an older age. Our study has the highest number of hospitals and population performed in our country and had some similarities in the results to other studies from developed countries [15–17].

Aging can influence the healing process of any wound due to several physiologic changes. Our study showed that an AL has 2.5 more odds to affect patients who are 65 years or older. This characteristic has been found to be a risk factor in several studies. For instance, Fabian *et al* [15] and Lin *et al* [18] who found similar results concluded that an age of 70 or older has an increased risk of AL. The physiologic explanation is that the healing process is slower due to physiologic changes that are associated with aging in elderly population. There is a decreased production and number of collagen fibres due to the lower activity of the enzyme collagenase and less angiogenesis at the vascular level [19]. Both processes impact on matric remodelling when the anastomosis happens. These processes do not only affect elderly population as a risk factor to develop this, also in clinical outcomes after the appearance and recovery [20].

A higher ASA score has been associated with worse short- and long-term clinical outcomes in colorectal cancer and other types of malignant neoplasms [21]. Our study found that AL has approximately 320% increase in the odds of affecting patients with an ASA score ≥III after colorectal surgery. Similarly, Buchs *et al* [22] determined that the risk of Al increased about 2.5 times for each point in ASA score. Furthermore, a study performed by Bakker *et al* [23] showed that patients with ASA III and IV had a higher occurrence of AL than patients with ASA I and II (9.2% versus 7.1%, *p* < 0.001). There are several local and systemic factors that influence on this complication, the adaptive changes produced by chronic comorbidities such as diabetes, cardiac diseases, tobacco, and peripheral vascular diseases can severely alter the postsurgical healing and haemostatic process.

One of the most important markers of nutrition is the albumin levels which is crucial for the recovery of any surgical procedure. In our population, malnutrition determined by low albumin levels was the most important predictor of DA, this complication occurred 22.2 more times in these patients. Flores Medina [24] and Fabian *et al* [15] demonstrated that lower albumin levels increased the risk of DA by 2.8 and 6.0, respectively. Moreover, a multicentric study performed by Frasson *et al* [25] showed there is a 30% decreased risk of DA for each g/dL higher. The association of DA and albumin is based on the decreased oncotic intravascular pressure which leads to tissue oedema and slow tissue healing, and the high demand of proteins to produce collagen fibres. The most likely reason for the higher risk in our population is because we included only colorectal cancer patients, which metabolic demand after surgical intervention is much higher than the other populations due to the oncologic process itself and postsurgical recovery. In addition to this, our population mainly consisted of patients with advanced stages, where nutrition is one of the most important factors in their immunity, which is a crucial factor for the development of AL.

The higher clinical stage of colorectal cancer is associated with a higher number of surgical complications and oncological prognosis of the patient [5, 26]. In our study, AL had 2.7 higher odds to occur in patients with AJCC stages III and IV. This finding was also found by Otiniano *et al* [16], who concluded that patients with T3–T4 had a 7.7 higher risk of developing AL. Similarly Pop *et al* [26], Rencuzogullari *et al* [5] and Xu *et al* [27] confirmed that there a higher clinical stage increases the risk of AL by 3.93, 1.42 and 10.34 times, respectively. This is explained by the worse physical performance, presence of cachexia, higher metabolic demands, more complex oncologic resection, and constant catabolic state that avoiding the optimal healing process of the anastomosis.

Despite several studies have shown other intra-, and peri-operative factors associated with AL such as the use of mechanical sutures, the surgical procedure in an emergency setting, shorter operative time, intraoperative blood loss and transfusion, histological margin involvement, surgical approach, between others [28–30]. Our results may vary from others due to the different population included, such as only colorectal patients, higher clinical stages, and the population itself was from national hospitals with scarce resources and high health care needs. The late stage at diagnosis has several causes with a high oncologic burden such as low coverage for cancer screening, inequalities due to socioeconomic status, inadequate resources, low level of education, not optimal care-seeking behaviour and limited access to screening and care [31–33]. Cancer screening and care with new health policies, plans, and campaigns are an urgent need in Latin America and developing countries [34].

Regarding the laterality of the resection, previous have shown that AL rates are usually higher in left colectomies with around 5% versus 1%–2% in right colectomies [35, 36]. Nevertheless, Hung *et al* [37] concluded in a study of 2,223 patients who underwent oncological resection that there was no difference in AL rates regardless of the laterality. Patients with left colorectal cancer, especially in a distal location, should be appropriately selected to optimise outcomes. Hence, there are several studies to evaluate the risk factors of AL but these are performed in all populations and not only in colorectal cancer patients, who differs completely from a non-oncological population [15, 24, 28, 38, 39]. It is crucial to perform further multicentric studies in colorectal cancer patients in this setting in order to prevent and have an earlier diagnosis to avoid the highly increased costs and length of stay in these patients [40].

Several institutions have been implemented pathways to improve outcomes during the last years such as ERAS protocols. Previous studies have shown that there are several benefits such as acceleration of recovery, effective analgesia, early oral feeding, ambulation, decreased costs and length of hospital stay, between others, without increasing and similar AL rates compared to the conventional pathway [41–43]. Currently, in Peru there are not formal ERAS protocols among most of the institutions, which could have a high impact on morbidity and mortality, but these would need to be adjusted to the health system, resources and infrastructure of the hospitals [44].

To date the current trend is to perform minimally invasive surgery for colorectal cancer patients, however, this is not the case in developing countries like Peru where most of the cases are still performed with an open approach [8, 10]. A meta-analysis by Song *et al* [45] concluded that laparoscopic surgery is superior to open in terms of estimated blood loss, hospital stay, postoperative mortality and complications. However, similar AL rates have been found between both approaches [46, 47]. Furthermore, it has been demonstrated that the laparoscopic approach is oncologically safe for colorectal cancer with similar disease-free and overall survival rates [48, 49]. Due to the several benefits of laparoscopic surgery, it is crucial to continue the surgical training of this technique and prioritise this approach when possible.

Our study has some limitations. Due to the design of the study as retrospective case control. The sociodemographic, clinical and surgical characteristics from some patients were incomplete, the reason for not being included in our study, which could have had some effect in our results despite we included a big sample avoid bias. Due to the inclusion of three national hospitals our results can be extrapolated to the population in Lima, capital of Peru, however, our results should be taken with caution when generalising Peruvian or Latin population in the setting that all the hospitals included in our study were in Lima. None of our patients had protective ileostomy or early endoscopy that some studies have shown to have a possible positive impact on the development of AL, these procedures can be useful to be evaluated in our population, especially among patients with higher clinical stages for prevention, early diagnosis, and treatment of this complication [28, 50]. Moreover, 55% of our patients had air leak test which could limit our results in patients who did not have a leak test, future studies may consider that all patients should undergo a leak test with air, endoscopy, or colonoscopy intraoperatively. We recommend better evaluation of the patients with advanced or metastatic colorectal cancer at diagnosis to determine better candidacy to undergo resection with anastomosis and provide optimal nutrition and treatment. Future studies might include other factors such as surgeon experience, pre-albumin, further details about the AL such as grading according to the AL consensus, and consequences in morbidity and mortality of these patients [11]. It would also be useful if the colon and rectum are separated as different identities [11]. Moreover, multicentric study with different hospitals and cities from Peru, Latin population and other developing countries should be performed to identify risk factors in a setting with scarce resources and elaborate predictive protocols of AL.

In conclusion, independent risk factors for AL in a setting of a developing country and high health care needs were pre-operative factors, while intraoperative and post-operative factors were not related to this complication. Patients with malnutrition were at most risk to develop this complication, most likely due to the high percentage of advanced tumour staging and poor physical performance status, for which pre-operative nutritional optimisation and adequate selection of patients should be standardised to improve clinical outcomes in colorectal cancer patients.

## Conflicts of interest

None of the authors have any commercial interest in the subject of this study that may be perceived as a potential conflict of interest. No industry or financial/material support was given specifically for this study.

## Funding

This research received no specific grant from any funding agency in the public, commercial, or not-for-profit sectors.

## Data availability

The data that support the findings of this study are available from the corresponding author upon reasonable request. The data that support the findings of this study are available from the corresponding author upon reasonable request.

## Author contributions

SLRR contributed to conceptualisation – ideas; data curation; methodology; validation; visualisation; writing – original draft; writing – review and editing.

DFSM contributed to conceptualisation – ideas; writing – original draft; writing – review and editing.

GDK contributed to conceptualisation – ideas; data curation; methodology;

validation; visualisation; writing – original draft; writing – review and editing.

CRLM contributed to conceptualisation – ideas; writing – original draft; writing – review and editing.

CRRB contributed to conceptualisation – ideas; writing – original draft; writing – review and editing.

## Figures and Tables

**Table 1.** Sociodemographic and surgical characteristics from patients who underwent surgery for colorectal cancer at three hospitals in Lima during 2021–2022.

Factor	Frequency (*n*)	Percentage (%)
Age		
<65 years	122	33.9
≥65 years	238	66.1
Sex		
Female	190	52.8
Male	170	47.2
Diabetes mellitus		
No	202	56.1
Yes	158	43.9
Tobacco		
No	335	93.1
Yes	25	6.9
AJCC stage^a^		
I	14	3.9
II	97	26.9
III	228	63.3
IV	21	5.8
ASA score^b^		
II	331	91.9
III	29	8.1
Albumin		
≥3.5 g/dL	216	60.0
<3.5 g/dL	144	40.0
Preoperative haemoglobin		
≥11 g/dL	200	55.6
<11 g/dL	160	44.4
Preoperative creatinine		
≤1.4 mg/dL	353	98.1
>1.4 mg/dL	7	1.9
Type of surgery		
Elective	347	96.4
Emergency	13	3.6
Surgical approach		
Laparoscopic	112	31.1
Open	248	68.9
Operative time		
≤180 minutes	69	19.2
>180 minutes	291	80.8
Anastomosis technique		
Handsewn	220	61.1
Mechanical	140	38.9
Type of anastomosis		
E-E^c^	102	28.3
E-L^d^	24	6.7
L-L^e^	234	65.0
Intraoperative transfusion		
No	346	96.1
Yes	14	3.9
Tumour location		
Right colon	183	50.8
Transverse colon	25	6.9
Left colon	102	28.3
Rectum	50	13.9
Time to restart diet		
≤3 days	33	9.2
>3 days	327	90.8

^a^AJCC, American Joint Committee on Cancer; ^b^ASA, American Society of Anesthesiologists; ^c^E-E, end-to-end anastomosis; ^d^E-L, end-to-lateral anastomosis; ^e^L-L, lateral-to-lateral anastomosis

**Table 2.** Sociodemographic and surgical characteristics from patients who underwent surgery for colorectal cancer in Lima during 2021–2022 according to the hospital. Statistically significant values are in boldface type.

Factors	‘Dos de Mayo’ National Hospital *n* (%)	‘Edgardo Rebagliati Martins’ National Hospital *n* (%)	‘Alberto Sabogal Sologuren’ National Hospital *n* (%)	*p* value
Age				
<65 years	51 (42.5)	35 (29.2)	36 (30.0)	**0.020**
≥65 years	69 (57.5)	85 (70.8)	84 (70.0)	
Sex				
Female	63 (52.5)	66 (55.0)	61 (50.8)	0.389
Male	57 (47.5)	54 (45.0)	59 (49.2)	
Diabetes mellitus				
No	71 (59.2)	62 (51.7)	69 (57.5)	0.262
Yes	49 (40.8)	58 (48.3)	51 (42.5)	
Tobacco				
No	108 (90.0)	110 (91.7)	117 (97.5)	0.094
Yes	12 (10.0)	10 (8.3)	3 (2.5)	
AJCC stage^a^				
I–II	27 (22.5)	38 (31.7)	46 (38.3)	0.329
III–IV	93 (77.5)	82 (68.3)	74 (61.7)	
ASA score^b^				
II	116 (96.7)	109 (90.8)	106 (88.3)	**0.024**
III	4 (3.3)	11 (9.2)	14 (11.7)	
Albumin				
≥3.5 g/dL	71 (59.2)	69 (57.5)	76 (63.3)	0.471
<3.5 g/dL	49 (40.8)	51 (42.5)	44 (36.7)	
Preoperative hemoglobin				
≥11 g/dL	73 (60.8)	67 (55.8)	60 (50.0)	0.185
<11 g/dL	47 (39.2)	53 (44.2)	60 (50.0)	
Preoperative creatinine				
≤1.4 mg/dL	117 (97.5)	117 (97.5)	119 (99.2)	0.757
>1.4 mg/dL	3 (2.5)	3 (2.5)	1 (0.8)	
Type of surgery				
Elective	111 (92.5)	118 (98.3)	118 (98.3)	0.486
Emergency	9 (7.5)	2 (1.7)	2 (1.7)	
Surgical approach				
Laparoscopic	84 (70.0)	27 (22.5)	1 (0.8)	**<0.001**
Open	36 (30.0)	93 (77.5)	119 (99.2)	
Operative time				
≤180 minutes	2 (1.7)	33 (27.5)	34 (28.3)	**<0.001**
>180 minutes	118 (98.3)	87 (72.5)	86 (71.7)	
Anastomosis technique				
Handsewn	98 (81.7)	17 (14.2)	105 (87.5)	**<0.001**
Mechanical	22 (18.3)	103 (85.8)	15 (12.5)	
Type of anastomosis				
E-E^c^	34 (28.3)	37 (30.8)	31 (25.8)	0.815
E-S^d^	10 (8.3)	7 (5.8)	7 (5.8)	
S-S^e^	76 (63.3)	76 (63.3)	82 (68.3)	
Intraoperative transfusion				
No	115 (95.8)	118 (98.3)	113 (94.2)	0.396
Yes	5 (4.2)	2 (1.7)	7 (5.8)	
Tumour location				
Right colon	49 (40.8)	59 (49.2)	75 (62.5)	**0.009**
Transverse colon	12 (10.0)	6 (5.0)	7 (5.8)	
Left colon	37 (30.8)	39 (32.5)	26 (21.7)	
Rectum	22 (18.3)	16 (13.3)	12 (10.0)	
Time to restart diet				
≤3 days	12 (10.0)	20 (16.7)	1 (0.8)	**<0.001**
>3 days	108 (90.0)	100 (83.3)	119 (99.2)	

^a^The American Joint Committee on Cancer; ^b^The American Society of Anesthesiologists; ^c^End to End; ^d^End to Side; ^e^Side to Side

**Table 3.** Bivariate analysis of sociodemographic and surgical characteristics from patients who underwent surgery for colorectal cancer in Lima during 2021–2022 according to the presence of AL. Statistically significant values are in boldface type.

Factors	AL		*p* value
	No *n* (%)	Yes *n* (%)	
Age			**0.003**
<65 years	94 (39.2)	28 (23.3)	
≥65 years	146 (60.8)	92 (76.7)	
Sex			0.502
Female	130 (52.2)	60 (50.0)	
Male	110 (45.8)	60 (50.0)	
Diabetes mellitus			0.071
No	143 (70.8)	59 (29.2)	
Yes	97 (61.4)	61 (38.6)	
Tobacco			0.125
No	227 (94.6)	108 (90.0)	
Yes	13 (5.4)	12 (10.0)	
AJCC stage^a^			**0.000**
I–II	90 (37.5)	21 (17.5)	
III–IV	150 (62.5)	99 (82.5)	
ASA score^b^			**0.013**
II	227 (94.6)	104 (86.7)	
III	13 (5.4)	16 (13.3)	
Albumin			**<0.001**
≥3.5 g/dL	194 (80.8)	22 (18.3)	
<3.5 g/dL	46 (19.2)	98 (81.7)	
Preoperative haemoglobin			0.432
≥11 g/dL	137(57.1)	63 (52.5)	
<11 g/dL	103 (42.9)	57 (47.5)	
Preoperative creatinine			1.000
≤1.4 mg/dL	235 (97.9)	118 (98.3)	
>1.4 mg/dL	5 (2.1)	2 (1.7)	
Type of surgery			0.556
Elective	230 (95.8)	117 (97.5)	
Emergency	10 (4.2)	3 (2.5)	
Surgical approach			0.630
Laparoscopic	77 (32.1)	35 (29.2)	
Open	163 (67.9)	85 (70.8)	
Operative time			0.320
≤180 minutes	50 (20.8)	19 (15.8)	
>180 minutes	190 (79.2)	101 (84.2)	
Anastomosis technique			0.819
Handsewn	148 (61.7)	72 (60.0)	
Mechanical	92 (38.3.7)	48 (40.0)	
Type of anastomosis			0.060
E-E^c^	60 (25.0)	42 (35.0)	
E-S^d^	19 (7.9)	5 (4.2)	
L-L^e^	161 (67.1)	73 (60.8)	
Intraoperative transfusion			0.564
No	232 (96.7)	114 (95.0)	
Yes	8 (3.3)	6 (5.0)	
Tumour location			**0.001**
Right colon	132 (55.0)	51 (42.5)	
Transverse colon	19 (7.9)	6 (5.0)	
Left colon	68 (28.3)	34 (28.3)	
Rectum	21 (8.8)	29 (24.2)	
Time to restart diet			0.847
≤3 days	23 (9.6)	10 (8.3)	
>3 days	217 (90.4)	110 (91.7)	

^a^The American Joint Committee on Cancer; ^b^The American Society of Anesthesiologists; ^c^End to End; ^d^End to Side; ^e^Side to Side

**Table 4.** Multivariate logistic regression analysis of sociodemographic and surgical characteristics from patients who underwent surgery for colorectal cancer in Lima during 2021–2022 according to the presence of AL. Statistically significant values are in boldface type.

Variables	AL			
	OR _crude_ (95%CI)	*p* value	OR _adjusted_ (95%CI)	*p* value
Age				
<65 years	Ref.	Ref.	Ref.	Ref.
≥65 years	2.11 (1.29–3.48)	0.003	2.48 (1.24–4.97)	**0.010**
Sex				
Female	Ref.	Ref.	Ref.	Ref.
Male	1.18 (0.76–1.83)	0.456	1.23 (0.67–2.27)	0.497
Diabetes mellitus				
No	Ref.	Ref.	Ref.	Ref.
Yes	1.52 (0.98–2.37)	0.061	1.47 (0.78–2.80)	0.229
Tobacco/alcohol				
No	Ref.	Ref.	Ref.	Ref.
Yes	1.94 (0.86–4.39)	0.112	1.23 (0.37–4.19)	0.731
AJCC stage^a^				
I–II	Ref.	Ref.	Ref.	Ref.
III–IV	2.82 (1.65–4.85)	0.000	2.71 (1.34–5.48)	**0.005**
ASA score^b^				
II	Ref.	Ref.	Ref.	Ref.
III	2.68 (1.25–5.79)	0.012	3.23 (1.10–9.50)	**0.032**
Albumin				
≥3.5 g/dL	Ref.	Ref.	Ref.	Ref.
<3.5 g/dL	18.7 (10.7–32.9)	0.000	22.2 (11.5–42.9)	**<0.001**
Preoperative hemoglobin				
≥11 g/dL	Ref.	Ref.	Ref.	Ref.
<11 g/dL	1.20 (0.78–1.87)	0.410	0.84 (0.46–1.58)	0.605
Preoperative creatinine				
≤1.4 mg/dL	Ref.	Ref.	Ref.	Ref.
>1.4 mg/dL	0.79 (0.15–4.17)	0.788	0.60 (0.04–9.52)	0.722
Type of surgery				
Elective	Ref.	Ref.	Ref.	Ref.
Emergency	0.59 (0.16–2.18)	0.429	0.59 (0.08–4.77)	0.626
Surgical approach				
Laparoscopic	Ref.	Ref.	Ref.	Ref.
Open	1.14 (0.71–1.85)	0.573	1.18 (0.59–2.36)	0.634
Operative time				
≤180 minutes	Ref.	Ref.	Ref.	Ref.
>180 minutes	1.39 (0.78–2.50)	0.257	1.32 (0.58–3.05)	0.506
Anastomosis technique				
Handsewn	Ref.	Ref.	Ref.	Ref.
Mechanical	1.07 (0.69–1.68)	0.760	1.10 (0.59–2.08)	0.758
Type of anastomosis				
E-E^c^	Ref.	Ref.	Ref.	Ref.
E-S^d^	0.37 (0.13–1.09)	0.071	0.32 (0.07–1.47)	0.141
S-S^e^	0.64 (0.40–1.05)	0.077	0.65 (0.27–1.63)	0.365
Intraoperative transfusion				
No	Ref.	Ref.	Ref.	Ref.
Yes	1.52 (0.51–4.50)	0.444	1.34 (0.28–6.55)	0.714
Tumour location				
Right colon	Ref.	Ref.	Ref.	Ref.
Transverse colon	0.81 (0.31–2.16)	0.685	1.29 (0.33–5.03)	0.714
Left colon	1.29 (0.77–2.18)	0.334	1.24 (0.56–2.80)	0.590
Rectum	3.57 (1.87–6.83)	0.000	3.05 (0.95–9.87)	0.062
Time to restart diet				
≤3 days	Ref.	Ref.	Ref.	Ref.
>3 days	1.16 (0.54–2.54)	0.699	1.08 (0.37–3.17)	0.882

^a^The American Joint Committee on Cancer; ^b^The American Society of Anesthesiologists; ^c^End to End; ^d^End to Side; ^e^Side to Side

## References

[1] Ferlay J, Ervik M, Lam F (2020). https://gco.iarc.fr/today.

[2] Stamos MJ, Brady MT (2018). Anastomotic leak: are we closer to eliminating its occurrence. Ann Laparosc Endosc Surg.

[3] Slieker JC, Daams F, Mulder IM (2013). Systematic review of the technique of colorectal anastomosis. JAMA Surg.

[4] He J, He M, Tang JH (2023). Anastomotic leak risk factors following colon cancer resection: a systematic review and meta-analysis. Langenbeck's Arch Surg.

[5] Rencuzogullari A, Benlice C, Valente M (2017). Predictors of anastomotic leak in elderly patients after colectomy: nomogram-based assessment from the American College of Surgeons National Surgical Quality Program procedure-targeted cohort. Dis Colon Rectum.

[6] Tsalikidis C, Mitsala A, Mentonis VI (2023). Predictive factors for anastomotic leakage following colorectal cancer surgery: where are we and where are we going?. Curr Oncol.

[7] Ortega Checa D, Vojvodic Hernandez IM, Pinares Carrillo D (2020). Resultados de la aplicación del Protocolo de Recuperación Mejorada en Cirugía (PREMEC) en el tratamiento quirúrgico del cáncer colorrectal en el Hospital Nacional Edgardo Rebagliati - EsSalud. Rev Gastroenterol Perú.

[8] Vaca-Bautista B (2024). Resultados Postoperatorios en pacientes con cáncer de colon sometidos a cirugía laparoscópica versus cirugía abierta en el HNERM de julio 2016 a julio 2021: Un estudio de cohorte retrospectivo.

[9] Fernandez-Concha P (2019). Eficacia de la cirugia laparoscopic colorrectal versus la cirugia colorectal abierta en la reduccion de la estancia postoperatoria.

[10] Guevara-Jabiles A, Berrospi F, Chávez I (2022). Open versus minimally invasive sphincter-sparing surgery for rectal cancer: a single-center retrospective cohort study in Peru. Rev Gastroenterol Perú.

[11] van Helsdingen CP, Jongen AC, Jonge WJ (2020). Consensus on the definition of colorectal anastomotic leakage: a modified Delphi study. World J Gastroenterol.

[12] Tsai YY, Chen WTL (2019). Management of anastomotic leakage after rectal surgery: a review article. J Gastrointest Oncol.

[13] Gessler B, Eriksson O, Angenete E (2017). Diagnosis, treatment, and consequences of anastomotic leakage in colorectal surgery. Int J Colorectal Dis.

[14] Stormark K, Krarup PM, Sjövall A (2020). Anastomotic leak after surgery for colon cancer and effect on long-term survival. Colorectal Dis.

[15] Fabian K (2012). Determinants of Intestinal Anastomosis Dehiscence at Ramiro Prialé Prialé National Hospital – Huancayo, from January 2010 to January 2012.

[16] Otiniano Alvarado C (2019). Risk Factors for Aanastomotic Dehiscence in Adult Patients After Low Anterior Resection of Rectal Cancer at the Instituto Nacional de Enfermedades Neoplásicas during the period 2009–2015.

[17] Wako G, Teshome H, Abebe E (2019). Colorectal anastomosis leak: rate, risk factors and outcome in a Tertiary Teaching Hospital, Addis Ababa Ethiopia, a five year retrospective study. Ethiop J Health Sci.

[18] Lin JK, Yueh TC, Chang SC (2011). The influence of fecal diversion and anastomotic leakage on survival after resection of rectal cancer. J Gastrointest Surg.

[19] Lenhardt R, Hopf HW, Marker E (2000). Perioperative collagen deposition in elderly and young men and women. Arch Surg.

[20] Zaimi I, Sparreboom CL, Lingsma HF (2018). The effect of age on anastomotic leakage in colorectal cancer surgery: a population-based study. J Surg Oncol.

[21] Park JH, Kim DH, Kim BR (2018). The American Society of anesthesiologists score influences on postoperative complications and total hospital charges after laparoscopic colorectal cancer surgery. Medicine (Baltimore).

[22] Buchs NC, Gervaz P, Secic M (2008). Incidence, consequences, and risk factors for anastomotic dehiscence after colorectal surgery: a prospective monocentric study. Int J Colorectal Dis.

[23] Bakker IS, Grossmann I, Henneman D (2014). Risk factors for anastomotic leakage and leak-related mortality after colonic cancer surgery in a nationwide audit. Br J Surg.

[24] Flores Medina L (2019). Risk Factors Associated to Anastomotic Leak Following Colorectal Surgery at a Regional Hospital in Trujillo.

[25] Frasson M, Flor-Lorente B, Rodríguez JL (2015). Risk factors for anastomotic leak after colon resection for cancer: multivariate analysis and nomogram from a multicentric, prospective, national study with 3193 patients. Ann Surg.

[26] Pop M, Iancu D, Fiţ AM (2018). Anastomotic leak after colon cancer surgery. A retrospective, single-center analysis of 631 patients. Hum Vet Med.

[27] Xu H, Kong F (2020). Malnutrition-related factors increased the risk of anastomotic leak for rectal cancer patients undergoing surgery. BioMed Res Int.

[28] Telem DA, Chin EH, Nguyen SQ (2010). Risk factors for anastomotic leak following colorectal surgery: a case-control study. Arch Surg.

[29] Oprescu C, Beuran M, Nicolau AE (2012). Anastomotic dehiscence (AD) in colorectal cancer surgery: mechanical anastomosis versus manual anastomosis. J Med Life.

[30] Gomes SL, Santos PMDD, Costa Pereira J (2023). Ileocolic anastomosis dehiscence in colorectal cancer surgery. Gastrointest Disord.

[31] Vallejos C (2013). National plan for prevention, early detection, and cancer control in Peru. Am Soc Clin Oncol Educ Book.

[32] Revilla-López J, Anampa-Guzmán A, Marquez LC (2019). Cancer cases detected in the prevention and control service of a private cancer clinic in Peru. Infect Agents Cancer.

[33] Bishehsari F, Mahdavinia M, Vacca M (2014). Epidemiological transition of colorectal cancer in developing countries: environmental factors, molecular pathways, and opportunities for prevention. World J Gastroenterol.

[34] Piñeros M, Laversanne M, Barrios E (2022). An updated profile of the cancer burden, patterns and trends in Latin America and the Caribbean. Lancet Reg Health Am.

[35] Suding P, Jensen E, Abramson MA (2008). Definitive risk factors for anastomotic leaks in elective open colorectal resection. Arch Surg.

[36] Veyrie N, Ata T, Muscari F (2007). Anastomotic leakage after elective right versus left colectomy for cancer: prevalence and independent risk factors. J Am Coll Surg.

[37] Hung L, Darabnia J, Judeeba S (2022). Timing and outcome of right- vs left-sided colonic anastomotic leaks: is there a difference?. Am J Surg.

[38] Garrido Fernández L (2023). Preoperative Factors Related to Anastomotic Leak in Adults at the Surgery Service at the Regional Hospital in Cajamarca: Period 2019–2021.

[39] Muñoz PN, Rodríguez GM, Pérez-Castilla A (2019). Evaluación de factores de riesgo asociados a dehiscencia anastomótica en cirugía colorrectal. Análisis multivariado de 748 pacientes. Rev Cirugía.

[40] Kang J, Kim H, Park H (2022). Risk factors and economic burden of postoperative anastomotic leakage related events in patients who underwent surgeries for colorectal cancer. PLoS One.

[41] Ota H, Ikenaga M, Hasegawa J (2017). Safety and efficacy of an "enhanced recovery after surgery" protocol for patients undergoing colon cancer surgery: a multi-institutional controlled study. Surg Today.

[42] Cavallaro P, Bordeianou L (2019). Implementation of an ERAS pathway in colorectal surgery. Clin Colon Rectal Surg.

[43] Eva P, Luca P, Manuela R (2024). Implementation of an enhanced recovery after surgery protocol for colorectal cancer in a regional hospital network supported by audit and feedback: a stepped wedge, cluster randomised trial. BMJ Qual Saf.

[44] Andrés Guevara J, Edith Elizabeth Cedeño A, Francisco Manuel Berrospi E (2021). Enhanced recovery after surgery in colorectal cancer. Instituto Nacional de Enfermedades Neoplásicas. Acta Med Peru.

[45] Song XJ, Liu ZL, Zeng R (2019). A meta-analysis of laparoscopic surgery versus conventional open surgery in the treatment of colorectal cancer. Medicine (Baltimore).

[46] Fleshman J, Branda M, Sargent DJ (2015). Effect of laparoscopic-assisted resection vs open resection of stage II or III rectal cancer on pathologic outcomes: the ACOSOG Z6051 randomized clinical trial. JAMA.

[47] Chen H, Ma B, Gao P (2017). Laparoscopic intersphincteric resection versus an open approach for low rectal cancer: a meta-analysis. World J Surg Oncol.

[48] Group TLAvOCTS (2007). Laparoscopically assisted vs open colectomy for colon cancer: a meta-analysis. Arch Surg.

[49] Bonjer HJ, Deijen CL, Abis GA (2015). A randomized trial of laparoscopic versus open surgery for rectal cancer. N Engl J Med.

[50] Ferko A, Rejholoc J (2021). Colorectal anastomosis dehiscence: a call for more detailed morphological classification. Videosurgery Other Miniinv Tech.

